# Plant-Based Food for the Prevention of Type 2 Diabetes: Scoping Review

**DOI:** 10.3390/nu16111671

**Published:** 2024-05-29

**Authors:** Jéssica Carolinne Damasceno e Silva, Isabele Christina Andrade Bezerra Anghinoni, Marília Brito Gomes

**Affiliations:** 1 Diabetes Unit, Department of Internal Medicine, State University of Rio de Janeiro, Vila Isabel, Rio de Janeiro 20551-030, RJ, Brazil; mariliabgomes@gmail.com; 2 Diabetes Unit, Department of Internal Medicine, Lagoa Federal Hospital of Rio de Janeiro, Lagoa, Rio de Janeiro 22470-050, RJ, Brazil

**Keywords:** type 2 diabetes, prevention, plant-based diet, glycemic control, cardiovascular risk

## Abstract

Type 2 Diabetes Mellitus (T2DM) is a chronic condition with growing worldwide prevalence. Besides genetic factors, a sedentary lifestyle, excess weight, and inadequate eating habits, characterized by an excess intake of refined carbohydrates and ultra-processed foods, are contributing factors for the development of the disease. In this scenario, promoting a plant-based diet, and limiting animal product consumption while increasing the intake of vegetables, concurrently with healthy lifestyle habits, is a promising strategy to prevent T2DM. This scoping review, carried out between 2017 and 2022, aimed to gather evidence substantiating the benefits of a plant-based diet in T2DM prevention, considering different eating patterns, such as vegetarian, vegan, Mediterranean, and DASH diets. Several studies demonstrate a significant reduction in T2DM incidence among individuals adopting plant-based eating patterns or emphasizing healthy plant-based food alongside decreased intake or exclusion of animal-based foods. There are still no robust data regarding plant-based diets and the prevention of diabetes without loss in body weight. Hence, prospective studies in plant-based diets with weight control are needed. Nevertheless, adopting plant-based diets appears to induce significant weight loss, which is crucial in an obesity-endemic context. Thus, embracing plant-based diets, along with healthy habits, emerges as a relevant strategy in obesity and T2DM prevention.

## 1. Introduction

Type 2 diabetes (T2DM) is a worldwide chronic disease. It is estimated that, in 2030, 578 million people will have diabetes, and this number will increase by 51%, affecting 700 million people by 2045.

According to the International Diabetes Federation (IDF), the etiology of T2DM has not been completely defined. However, it is clear that individuals with excess weight, unhealthy food habits, sedentarism, and a family history of diabetes present an increased risk of developing the disease [1,2]. With the understanding that unhealthy lifestyles can accelerate the appearance of diabetes, early intervention in food habits becomes one of the pillars of prevention.

In the last few years, the number of individuals adopting plant-based food patterns has increased. These patterns emphasize plant-based food and include a reduction in the intake, or even the exclusion, of animal-based food with the aim of preventing or controlling chronic diseases such as T2DM, cardiovascular disease, and cancer [2]. We know that excess weight, as well as obesity, is connected to the development of T2DM [2]. A randomized study observed that the function of beta cells and insulin sensitivity were significantly improved with a plant-based diet with low-fat content in adults with excess weight [3].

Poor nutrition comprises a variety of problems related to food intake, going beyond obesity and malnutrition, and can contribute to the development of non-transmissible chronic disease, hence becoming an important issue for public health. In addition, there is a growing concern about the impact of the food system on the environment and climate change. A plant-based diet is more sustainable since it cooperates with the reduction in greenhouse gas emissions [4].

Inflammation, along with insulin resistance, can be found in the pathogenesis of obesity, and both are related to the development of T2DM [5]. A plant-based diet is generally rich in fiber, chlorogenic acids, certain amino acids, unsaturated fatty acids, and antioxidants that can improve the level of inflammatory markers. On the other hand, animal protein, due to its rich content of branch chain amino acids and aromatic amino acids, can affect glucose metabolism, leading to alterations that contribute to hepatocellular oxidative stress. This interferes in the storage and utilization of glycogen, the insulin signaling pathway, and gluconeogenesis, all of which, when activated, are associated with an increase in the development of T2DM [5]. Furthermore, an animal-based diet is also rich in heme iron and other nutrients found in processed red meat, such as sodium and nitrates, and are factors that have been associated with cardiometabolic disease [5]. Wittenbecher et al. [5] studied the total intake of red meat associated with the evaluation and concentration of biomarkers in the blood. A total of 13 of the biomarkers found were significantly associated with the risk for diabetes, and 6 of the 13 (ferritin, glycine, diacyl-phosphatidylcholines, lysophosphatidycholine, and hydroxylated sphingomyelin) are consistently associated with the total intake of red meat and the risk for diabetes.

A meta-analysis published in 2019 that includes 185 prospective studies and 58 clinical trials, observed that for every 8 g of dietary fiber consumed per day, there was a 19% reduction in the prevalence of coronary atherosclerotic disease and 15% in the incidence of T2DM [6].

The term “plant-based diets” encompasses several food patterns that contain lower quantities of animal-based products and greater quantities of plant products, such as vegetables, fruit, whole grains, legumes, nuts, and seeds [7]. The different types of plant-based diets are described in Table 1.

Due to the increase in chronic diseases and the constant recommendation of a low intake of red meat and a high intake of plant-based foods by the main dietary guidelines, the main purpose of this scoping review is to collect evidence described in the literature to justify that a plant-based diet is beneficial in the prevention of T2DM. With this, we may see more consistent nutritional interventions regarding their benefits and adverse effects.

## 2. Materials and Methods

For this scoping review, electronic databases, such as PUBMED, Virtual Health Library (Biblioteca Virtual em Saúde—BVS), and SCIELO, were used to select articles. With the use of the tools pertaining to these databases, a list of published articles was obtained with the following words in English: “diet”, “prevention”, and “diabetes”.

**Figure 1:**
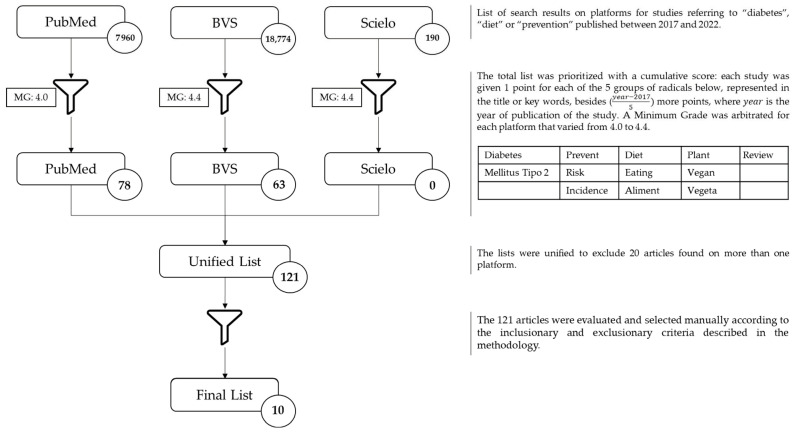
Selection flow of articles for review.

In a flow described in Figure 1, using the keywords mentioned above, an initial search yielded 26,924 articles. Subsequently, the methodology employed in this review involved the evaluation of an article database, assigning a score from 1 to 5 to each occurrence of word groups related to the topic under investigation. The aim of this approach was to facilitate the identification and selection of relevant studies by means of an analysis of abstracts, giving more weight to those with higher scores. Following this, a filtering process was applied, which resulted in 141 articles that met the criteria of a minimum grade of 4.0 for PUBMED articles and 4.4 for articles from other sources. After removing 20 duplicate articles, 121 articles remained for a more detailed analysis, consisting of reading of titles and abstracts, as well as the application of specific eligibility criteria for inclusion in this investigation.

Two authors independently analyzed the 121 articles, following predefined criteria. The inclusion criteria were (1) topic of investigation: studies that explored the relationship between plant-based diets and the prevention of T2DM; (2) language and period of publication: studies published in English between 2017 and 2022; (3) types of study: cohort-type studies, randomized clinical trials, systematic reviews, or meta-analyses. The studies were also required to focus on patients with no previous diagnosis of T2DM; (4) age group: studies with individuals over 18 years old, in other words, adults; (5) gender: studies encompassing all genders; (6) geographical area: without intentional geographic delimitation, allowing the inclusion of studies carried out in different regions around the world, in order to include different ethnic groups and guarantee a broader representation of population diversity. Consequently, studies that did not meet all these inclusion criteria established were excluded from the review.

To ensure consistency and the reliability of the selection, the authors had discussions to align their decisions and resolve possible divergences. At the end of the process, 10 articles were chosen that met the inclusion criteria previously established in this review.

This review was based on the evaluation criteria outlined by the PRISMA-ScR guidelines (Preferred Reporting Items for Systematic Reviews and Meta-Analyses extension for Scoping Reviews) to ensure a methodological accuracy that is aligned with the best scoping review practices. The final protocol was registered prospectively with the Open ScienceFramework on 26 March 2024 (https://osf.io/7ymg9, accessed on 17 May 2024).

## 3. Results

In total, 10 prospective studies, carried out with different ethnicities, met all the inclusionary criteria established, and investigated several food patterns associated with plant-based diets. These studies consistently showed a significant reduction in the risk of T2DM progression, as well as a reduction in body weight, according to what is described in Table 2.

## 4. Discussion

In the literature, the term “plant-based diet” has been associated with several food patterns such as vegetarianism, veganism, the Mediterranean diet, the dietary approach to stop hypertension (DASH), as well as with individuals who eat animal-based products, such as dairy, and others who allow a moderate quantity of red meat and totally exclude ultra-processed foods. All of these have demonstrated a reduction in the risk for chronic diseases such as T2DM, obesity, cardiovascular disease, and some types of cancer [19,20].

Plant-based diets are different from animal protein-based diets in how they act on intestinal microbiota and on their metabolic pathways, which includes an increase in fiber and polyphenol metabolism and a decrease in the metabolism of biliary acids and amino acids [19].

The gut microbiome has been evaluated as an influencer in cardiovascular risk. There is some evidence that compounds derived mainly from animal sources are related to a higher risk of cardiovascular events, independently of traditional risk factors. It is assumed that these compounds influence heart health through their effects on cholesterol and sterol metabolism, inflammation, and thrombotic and atherosclerotic pathways [19].

As an example, we have saturated fatty acids, very common in animal protein-based diets, which can interact with the intestinal microbiome and promote the translocation of lipopolysaccharides (LPS), a pro-inflammatory endotoxin, to the bloodstream. On the other hand, there is evidence that the polyunsaturated fatty acids found in plant-based diets activate anti-inflammatory pathways [19].

However, larger and more long-term studies, as well as metabolome and microbiome research, are needed to elucidate the complex mechanisms through which diet interacts with the gut microbial environment to impact cardiovascular health and other pro- and anti-inflammatory metabolic pathways [19].

The Plant-Based Diet Index (PDI) was developed by Satija et al. to evaluate the degree of adherence to different types of plant-based diets (healthy and unhealthy) and their association with the risk for T2DM progression based on a prospective study of a Caucasian population of more than 200,000 health professionals during 20 years in the United States [21]. The more predominant the intake of plant-based foods, the greater the PDI score. The diet base evaluated by the PDI consists of a pro-vegetarian diet with a greater intake of plant-based foods and a very low intake of animal-based foods [19].

Based on this, healthy and unhealthy versions of PDIs (hPDI, and uPDI, respectively) were put together with the same features. For example, fruit, legumes, whole wheat grains or vegetable oils, and tea/coffee were considered healthy PDIs, while refined grains, fruit juices, sweet beverages, potatoes, and sweets/desserts were tallied as having an unhealthy PDI. The results of this study provided support for the 2015 food guidelines in the US [21].

In the study by Satija et al., diets with a high Plant-Based Diet Index of healthy vegetables, or hPDI, were inversely associated with a risk for the incidence of T2DM. The association between the lower incidence of T2DM and the higher intake of plant-based diets in general, whether healthy or unhealthy, was considerably weakened when we computed categories of body mass index (BMI), while those that were only hPDI remained practically unaltered. The uPDI was positively associated with a greater incidence of T2DM, even after adjusting for BMI, which demonstrates the importance of plant quality [21].

Randomized clinical trials demonstrate the beneficial effects of diets that are rich in viscose and soluble fibers in improving post-prandial glucose levels, as well as in long-term glucose metabolism [21]. Thus, a healthy plant-based diet can improve glycemic control, and insulin sensibility, and diminish chronic inflammation, hence reducing the risk of chronic disease progression, principally T2DM. Furthermore, the high concentration of fiber and the low-calorie concentration found in many vegetables can reduce the development of T2DM even further, since these help in weight loss/maintenance [21].

Associations between changes in the quality of plant-based diets for 12 years (from 1986 to 1998) were investigated by Baden et al. in an adult Caucasian population in three prospective studies (evaluated by three indexes—an index for a general plant-based diet (PDI), an index for a healthy plant-based diet (hPDI), and an index for an unhealthy plant-based diet (uPDI), besides subsequent general mortality and specific cause from 1998 to 2014) [13].

The study surmised that the improvement in the quality of the healthy plant-based diet in a 12-year period was associated with a lower risk for general mortality and cardiovascular disease; and that an increase in the intake of an unhealthy plant-based diet was associated with a higher risk for general mortality and cardiovascular disease [13]. In the same prospective study mentioned above, Chen et al. [15] followed and observed that, in a Caucasian population, greater adherence to healthy plant-rich diets for four years was associated with a lower risk of T2DM progression in the four subsequent years, while a decrease in adherence to these diets was related to a higher risk of T2DM progression in 12 to 23% (grouped HR, PDI 1.12 [IC 95% 1.05, 1.20], hPDI 1.23 [1.16, 1.31]). The robustness of the results in Chen et al. in the sensibility analyses suggests that improving adherence to healthy plant-based diets can reduce the risk of T2DM progression, regardless of the basal quality of the diet. In other words, it was clear that even if an individual starts with a low PDI score, the consumption of healthy plant-based foods leads to discernible changes, highlighting the enduring benefits of maintaining such a dietary pattern. This has important implications for public health since future nutritional public policies should consider the quality of plant-based foods in reducing the risk of chronic disease progression [13].

In the study with the Korean Asian population [14], the prospective association between the scores of three different plant-based diet indices and the risk of T2DM progression was explored, while also investigating if the associations were different due to the demographic and lifestyle factors of the Korean population. A 10-point higher score in the hPDI was associated with a 14% lower risk of T2DM progression (HR: 0.86, IC 95%, 0.77–0.95), even after adjustments for possible misleading factors (sex, basal BMI, a family history of diabetes and of high blood pressure). In the subgroup analyses, the inverse relation was stronger among participants with a family history of T2DM or high blood pressure. However, plant-based diets in general (gathered by the PDI) and unhealthy plant-based diets (gathered by the uPDI) were not significantly associated with T2DM progression [14].

A prospective cohort study carried out in a Taiwanese Buddhist population investigated the risk of developing T2DM in individuals who followed a vegetarian diet, and those who went from a non-vegetarian food pattern to a vegetarian pattern. Results showed that both types of diets were associated with a significant reduction of approximately 53% in the risk of T2DM progression. This risk reduction was consistent regardless of gender, the presence of metabolic syndrome, HDL-C levels, and fasting glucose levels. While, before T2DM, pathogenesis in Caucasians was predominantly attributed to obesity and insulin resistance, new evidence suggests that β cell dysfunction may be more important in the pathogenesis and more predictive for T2DM progression in Asians. For example, studies show that Japanese individuals with a normal tolerance to glucose have an insulin secretion capacity that is similar to Caucasian patients with diabetes. These psychopathological differences highlight the need for research on specific preventive strategies for this Asian population [18].

In another rural prospective study of the Chinese Asian population by Henan [11], it was verified that a higher score in the general plant-based diets was associated with a lower risk of T2DM progression [OR (IC 95%) when compared to extreme quartiles: 0.88 (0.79–0.98)]. A 1 SD (standard deviation) increase in the PDI was correlated to a reduced risk of 4% in T2DM progression. The inverse relation observed continued in the analyses of stratified subgroups by sex, age, BMI, educational level, tobacco use, alcohol use, and physical activity, though it was high among participants who had a lower monthly income per capita. In this study, there are interesting characteristics such as sample size and ethnicity (Chinese), which is usually less evaluated. Hence, we can conclude that the benefit of a plant-based diet can be observed in different ethnic groups. A limitation of the abovementioned study was that causality could not be determined since it was a transversal study. The presence of other healthy habits such as the practice of physical activity, which was not evaluated, might have influenced the results obtained.

On the other hand, a large prospective study of a Caucasian-based population carried out in Holland [12] observed that a diet with a larger intake of plants and a lower intake of animal-based foods was associated with lower insulin resistance and, consequently, with a reduced risk of pre-diabetes and T2DM progression. This suggests that a plant-based diet presents a protection factor against the development of these clinical conditions when compared to a diet that is rich in animal products [15]. In this study, the aim of the authors was to investigate if less healthy plant-based foods contributed to the observed associations. The associations between the less healthy plant-based diets and insulin resistance and the risk of pre-diabetes and T2DM progression were examined. There was evidence that the beneficial associations were the result mainly of the greater intake of healthy plant-based food groups and the lower intake of unhealthy animal and plant-based food groups. This corroborates the importance of considering the quality of the plant-based foods consumed. Furthermore, estimates for the plant-based food index remain similar after the exclusion of sugary beverages or less healthy combined plant-based foods, which shows that the results found were stable in several plant-based diet versions.

In the PREVIEW [16] study, data from four prospective studies in Caucasian populations, three European and one Canadian, were evaluated. This study, which includes 78,851 participants, observed that, after adjustments for lifestyle and dietary factors, a greater intake of proteins was associated with a lower risk of pre-diabetes and T2DM progression. The separate analysis of plant and animal protein intake indicated that these associations were significantly connected to the intake of plant protein. The replacement of 2% of plant protein in detriment of carbohydrates, but not fat, significantly increased the risk of pre-diabetes and T2DM progression. However, the associations mentioned above were not significant after adjusting for BMI and abdominal circumference, which demonstrates the significance of excess weight in the connection between protein intake and T2DM progression.

The presence of protein in diets has a potentializing effect on insulin activity, besides also promoting its secretion, which leads to the body’s increase in the utilization of glucose [16]. Besides this, both hypocaloric and protein-rich diets demonstrated greater weight loss and fat loss than those seen in diets with a low protein content. Since being overweight and obesity are possibly the most important risk factors for T2DM progression, these data indicate the reason that dietary proteins may have a role in preventing diabetes. It is important to emphasize that the results in the literature are still conflicting, as well as those concerning weight loss with protein-rich diets [16].

The DASH diet (Dietary Approaches to Stop Hypertension) [3], which includes the intake of fruit, vegetables, non-fat/low-fat dairy products, whole wheat grains, nuts and legumes, and limits saturated fats, cholesterol, red and processed meats, sweets, added sugars, salt, and sugary beverages, was developed by studies of the National Health Institute in the United States with the purpose of reducing blood pressure with the use of this diet alone.

The benefit that the DASH food pattern brings to cardiovascular health can be explained by the positive biological effects of the several essential nutrients found in abundance in the foods featured in this pattern. Nutrients such as magnesium and potassium must be highlighted, besides phytochemicals, including flavonoids, which have been shown to have anti-inflammatory and antioxidant properties [3].

Furthermore, it was observed that an increase in the intake of nitrate-rich foods, such as fruit and plants, especially leafy plants, may be associated with reduced blood pressure. This effect is explained by the role inorganic nitrates have in the non-enzymatic generation of nitric acid. Additionally, many of the foods recommended by the DASH diet have led to evidence of lower blood pressure and body weight in systematic reviews and meta-analyses of controlled trials, both in individuals with and without diabetes [3,22].

In a broad review of five prospective studies [3]—four of which were in the USA (with a population composed of White, Hispanic, and Black individuals) and one in Europe—of systematic reviews and meta-analyses on cardiometabolic outcomes, the DASH diet was associated with a significant decrease in the risk of T2DM progression (RR: 0.82; IC 95%: 0.74, 0.92), as well as a significant decrease in cardiovascular, coronary, and cerebral vascular disease progression. However, it was observed that in Caucasian American groups, there was a strong inverse association between the DASH score (a high intake of the DASH food pattern) and the incidence of T2DM, which was not observed among Blacks and Hispanics. On the other hand, there was no decrease in the risk of T2DM progression observed in the European population in countries such as France, Germany, Spain, and Sweden.

In the data analysis in this study, the changes observed in fasting insulin levels and in the HOMA-IR index were considered moderate after statistical adjustments. Considering that the HOMA-IR is a calculation used to evaluate the degree of insulin resistance when there is high insulin resistance, we have peaks in insulin production in the attempt to maintain blood glucose levels within normal limits. A plant-based diet can contribute to the stabilization of glycemic values, generally keeping them lower, which would lead to lower HbA1C levels through time, and explains the decrease in HbA1C observed in this study [3].

The DASH diet, which features a high intake of foods rich in viscous fibers and whole wheat grains, has been associated with a lower impact on the increase in insulin in the HOMA-IR index, according to reviews conducted in the study by Chiavaroli et al. This observation suggests that the DASH diet may contribute to a lower glycemic variability and, possibly, to a reduction in HbA1C levels due to the abovementioned effects. However, it is important to highlight that estimates of this effect are still uncertain for most glycemic results, which means that there is a need for broad, high-quality randomized trials to clarify the benefits of glycemic parameters [3].

Other data such as lipid profile, inflammatory markers, blood pressure, and abdominal circumference were also utilized by some authors to predict metabolic syndrome, the level of anti-inflammatory or pro-inflammatory action, the relationship with adiposity, and cardiovascular risk. The results showed heterogeneity, with some studies providing evidence of improvement in these parameters with adherence to a plant-based diet compared to non-significant values or worsening with an animal-based diet. However, adjustments and more robust studies are needed for a complete understanding [3,14,16].

Other types of diet, such as the Mediterranean, which consists of the daily intake of plants, a variety of minimally processed whole wheat breads and other cereals and legumes, such as nuts and seeds; oil as the main source of fat; a low or moderate intake of dairy products; a moderate intake of fish, fowl, and eggs; allowing the intake of red meat (once a week) and the moderate intake of wine, normally during meals, was tested for the prevention and treatment of T2DM [10]. This type of diet was considered superior to those with a low-fat content for long-term weight loss. A prospective cohort study carried out for 25 years among more than 25,000 Caucasian female health professionals in the US indicated that the greater intake of a Mediterranean diet was associated with a 30% lower progression of T2DM. In addition, the high scores for this diet were associated with lower biomarkers for insulin resistance [10] (adiposity, lipoprotein metabolism, and inflammation).

A Mediterranean diet enriched with cold-pressed extra-virgin olive oil, or nuts was shown to be effective in preventing T2DM in the elderly with high cardiovascular risk. This approach reduced the risk by 52% when compared to a low-fat diet. This may be partially due to an increase in the activity of a peptide similar to glucagon (GLP-1), a hormone that stimulates insulin production and inhibits glucagon secretion. The polyunsaturated fatty acids found in cold-pressed extra virgin oil have a crucial role in this process since they bond and stimulate the G protein-coupled receptors in enteroendocrine cells, which increase the production of GLP-1. This stimulates insulin release, resulting in a reduction in post-prandial hyperglycemia [20].

Adherence to this type of diet may influence mechanisms related to T2DM pathogenesis, which includes anti-inflammatory/antioxidant activity promoted by the presence of polyunsaturated fatty acids. The diet offers a variety of nutrients and metabolites that, when combined, have synergic effects in the primary and secondary prevention of T2DM [20].

In a recent meta-analysis [17] whose goal was to evaluate the prevalence of T2DM in vegans, five prospective studies were included. Among these, the Adventist Health Study included 40,000 participants of different non-Black ethnicities (non-Hispanic Caucasian, Hispanic, Eastern, Asian, Native Hawaiian or Pacific Islander, and Native American), and Black ethnicities (African-American, Caribbean, African, and others). It was observed that a vegetarian diet could protect against T2DM progression in both ethnic groups (OR 0.429, IC 95% 0.249–0.740 and OR 0.381, IC 95% 0.236–0.617, respectively).

The American Heart Association (AHA) recommends the intake of healthy protein sources, especially plants, such as soybeans, grains, lentils, chickpeas, and peas, to reduce cardiovascular risk. It has also indicated the replacement of animal-based foods for whole wheat plant-based foods, which have additional benefits for the health of the planet. However, a sustainable food pattern is not associated with a lower cardiovascular risk since a plant-based diet that is rich in refined carbohydrates and the addition of sugar can increase the risk of T2DM progression and cardiovascular disease. This reinforces the importance of choosing healthy plant-based foods [23].

It is important to highlight that individuals who choose a plant-based diet and, especially, those who completely restrict animal-based foods, such as vegans, are prone to nutritional deficiencies if they do not have adequate nutritional follow-ups with a distribution of macronutrients and micronutrients. Among these deficiencies are protein, vitamin B12, calcium, iron, zinc, Omega 3, and vitamin D, which should be monitored and supplemented periodically.

Regarding BMI, the data may underestimate the true associations due to the adjustment for BMI in statistical analyses. In plant-based dietary patterns, changes occur with BMI adjustment, which can act as both a confounder and a mediator. Several interventional and observational studies have indicated that increased consumption of plant foods can lead to short-term weight loss or long-term prevention of weight gain. It is likely that a considerable proportion of the protective association between plant-based diets and the risk of T2DM can be attributed to weight control. At the same time, small-scale intervention studies have demonstrated that plant-based dietary patterns improved glycemic control measures, regardless of body weight, for individuals with and without T2DM, suggesting that the health benefits extend beyond weight control. Furthermore, plant-based diets may also improve the profile of adiposity-related risk markers, including leptin, adiponectin, high-sensitivity *C*-reactive protein, and interleukin-6 [2,24].

## 5. Conclusions

T2DM is a multi-factor disease with a significant genetic component that has not been totally identified. Given the growing number of individuals worldwide with this disease, who present a risk of progression to chronic complications with potentially high costs for the public health system, it is important to act in adopting preventive measures.

A plant-based diet may be important not only to prevent T2DM and obesity and to improve other cardiovascular risk factors (high blood pressure and dyslipidemia), but also to ease the impact on the environment. A sustainable diet in which animal-based products, especially red meat and milk/dairy products, are replaced by plant-based products, has the potential to reduce greenhouse gas emissions [25].

In this review, we observed that there is some evidence of a reduction in the risk of T2DM progression, regardless of ethnicity, in individuals who have healthy food habits. Concomitantly, the studies suggest that a healthy plant-based diet promotes weight loss, which is associated with a reduced risk of developing T2DM. However, we must highlight that the data of most studies concerning diets and the practice of physical activities were self-reported, which may lead to imprecise results.

A significant portion of the observational studies published to date have relied on self-reported physical activity levels. Studies utilizing objective measures of energy expenditure associated with physical activity generally have limited sample sizes, hindering the adequate assessment of health benefits across various physical activity modalities [26].

There is evidence that aerobic training increases insulin sensitivity and improves vascular function, among other benefits such as increased aerobic fitness and reduced body fat. Resistance training offers significant benefits for glycemic control, in addition to improving muscle strength, bone density, and muscle quantity and quality. However, there are challenges in interpreting these data, as studies often do not detail how these adjustments were made or simply assume them as a confounding factor [26].

We must point out, therefore, that some limitations in these studies, such as the dietary information, were evaluated by means of self-reporting; hence, assessment errors cannot be discarded. Since many of these studies were observational, misleading factors may have influenced results. As an example, we can suppose that an individual who follows a plant-based diet can simply be adopting a healthier lifestyle (they are nonsmokers, physically active, of average weight), or they are healthier in general, which may be a possible explanation for this observation. However, in general, the authors adjusted important misleading factors.

Considering that we are experiencing an obesity epidemic, joining forces to encourage weight loss is paramount. A higher intake of dietary fiber, present in plant-based diets, can contribute to weight loss, since fiber-rich foods require more time to be chewed and promote gastric distension, triggering signs of satiety and slowing digestion. Also, the delayed absorption of nutrients can delay hunger and the subsequent intake of energy. However, for greater clarification of the connection between a plant-based diet and the prevention of diabetes without weight loss, prospective studies about plant-based diets with weight control are needed.

With the constant changes occurring in the food habits of the world population and the growing wave of people in search of foods with more plant products, and a low intake of red meat and animal-based products, it is important still to consider the quality of plant products, which can influence the development of chronic disease, mainly among individuals with risk factors.

## Figures and Tables

**Table 1:** Specification of different types of diets accentuating plants and related references [8,9,10,11].

Diet	Simplified Description
Vegan	Excludes all animal-based products [8,9].
Whole-food plant-based diet	Excludes highly processed foods, focusing on food in its most natural form and reducing animal-based food [8,9].
Raw vegan	Excludes all animal-based products, as well as products that cannot be eaten raw [8,9].
Lacto-ovo-vegetarian	Excludes all forms of meat, but accepts other animal-based products (dairy, eggs, honey) [8,9].
Lacto-vegetarian	Excludes all forms of meat and eggs but accepts, dairy, and honey [8,9].
Ovo-vegetarian	Excludes all forms of meat and dairy, but accepts eggs, honey, etc. [8,9].
Pescetarianism	Excludes meat, but accepts fish, dairy, eggs, honey, etc. [8].
Semi-vegetarian/flexitarian	Alternates between vegetarian diets and meat-based diets; consists mainly of vegetarian, with a minimum intake of meat [8].
DASH	Limits the intake of red meat and ultra-processed food [3].
Mediterranean	Limits the intake of red meat and processed food, accepts the moderate intake of fowl and fish; high intake of oil [10].

**Table 2:** Comparative summary of reviewed studies.

Author/Year	Population	Participants	Study Design	Duration	Objective	Results
Chen et al. (2018) [12]	Caucasian	6798 adults	Prospective	5 to 7 years	To investigate the level of adherence to a plant-based diet versus an animal-based diet and its association with pre-DM and T2DM insulin resistance.	Lower incidence of T2DM in a plant-based diet. After adjustment to BMI, risk remained significant for lower risk for T2DM.
Baden et al. (2020) [13]	Caucasian	121,700 nurses 51,529 male health professionals	Prospective	12 years	Associations of changes in plant-based diets and total mortality due to specific reason.	To improve quality of diet: lower risk of total death by CVDs. The higher the hPDI, the lower the weight gain.
Kim et al. (2022) [14]	Korean	3466 men 3927 women	Prospective	14 years	Association of 3 scores of plant-based diet indices and T2DM risk.	The higher the hPDI, 14% lower was the risk of T2DM. Participants had a lower BMI.
Yang et al. (2021) [11]	Chinese	37,985	Prospective	2 years	To evaluate if a plant-based diet is related to a lower risk for T2DM among adult Chinese.	The higher the PDI, the lower the risk for T2DM. Higher impact for BMIs > 24
Chen et al. (2021) [15]	Caucasian	763,468 women (Nurses’ Health Study-NHS) 81,569 women (NHS II) 34,568 men (HPFS)	Prospective	4 years	Association of changes with plant-based diets and risk for T2DM	Improvement of adherence to diet with time leads to lower risk for T2DM. The higher the hPDI, the lower the weight gain, and, subsequently, the lower the risk for T2DM.
Sluik et al. (2019) [16]	Caucasian	78,815 participants	Prospective	30 years on average	Prospective associations between the ingestion of total protein, plant, and animal, and the risk of pre-diabetes and diabetes in 4 populational studies included in the PREVIEW project.	The higher the intake of plant protein, the lower the risk for T2DM; part of the effect is explained by association with lower BMIs.
Ahmad et al. (2020) [10]	Caucasian	25,317 women	Prospective	20 years	To characterize the relative contribution of conventional and new biomarkers in reducing the risk for T2DM associated with a Mediterranean diet (MED) in a population in the US.	The greater intake of a MED diet reduces the risk for T2DM by 30%. Results were attenuated in women with BMI > 25 kg/m^2^.
Pollakova et al. (2021) [17]	Varied, with several Black and non-Black groups.	40,000 participants (Adventist Health Study)	Meta-analysis (Prospective)	2 years	To evaluate the relation between vegan diets and the risk for T2DM	Lower risk for T2DM in vegans. After adjusting BMI, the risk remained lower, and the average of BMI in vegans was 23.6 kg/m^2^.
Chiavaroli (2019) [3]	Caucasian	942,140 participants	Meta-analysis (Prospective)	5 to 20 years	Five prospective studies sought to evaluate the relation between the intake of the DASH food pattern and the incidence of T2DM	DASH food pattern reduces the risk for T2DM. Associated with weight loss.
Chiu et al. (2018) [18]	Caucasian	4626 participants	Cohort (Prospective)	5.2 years	To investigate the association between a vegetarian diet, changes in food patterns, and risk for diabetes in a Taiwanese Buddhist population.	A vegetarian diet reduced the risk for T2DM by 35% and found a 53% risk reduction in transitioning from a non-vegetarian to a vegetarian pattern, already adjusted to basal BMI.

Notes: PDI—general score index for plant-based diet. hPDI—score index for plant-based diets considered healthy. T2DM—type 2 Diabetes Mellitus. DASH—Dietary approaches to stop hypertension. BMI—Body Mass Index.

## Data Availability

The used datasets and/or analyzed during the current study are available with the corresponding author upon reasonable request due to privacy reasons.

## Abbreviations

IDF	International Diabetes Federation
T2DM	Type 2 diabetes
